# 

**Published:** 1893-09

**Authors:** 


					﻿ESTABLISHED I85S.	SUBSCRIPTION. $2.00. i
ATLANTA v;
MEDIC AU AND SURGICAL
JOURNAL.	*
new?eRrS.voLi1xXVIJ Atlanta, Ga , September, 1893. No. 7^
LUTHER B. GRANDY, M. D.,	M. B. HUTCHINS, M. D.,
MANAGING EDITOR.	•	Bl’bl NESS MANAGER.
				

